# An integrative neurogenomics workflow for precision medicine in neurodegenerative disorders

**DOI:** 10.3389/frdem.2026.1745504

**Published:** 2026-02-04

**Authors:** Carlos Perezcano, Mariana Pérez-Coria

**Affiliations:** 1 Center for Research in Precision Medicine and Clinical Genomics, GENOMICS 360, Mexico City, Mexico; 2 Universidad Contemporánea de las Américas, Morelia, Michoacán, Mexico

**Keywords:** Alzheimer, dementia, genomics, integrative workflow, neurodegenerative diseases, precision medicine

## Abstract

Neurodegenerative diseases represent an expanding global health challenge, with rapidly increasing prevalence and substantial economic impact. The therapeutic clinical approach continues to seek solutions through pharmacological means—such as inhibitors and antibodies—which, while sometimes controlling symptoms, have not addressed the underlying pathophysiology. By integrating advanced genomics with selected biochemical markers, under the continuous oversight of a multidisciplinary team working in consensus, it is possible to achieve a more comprehensive understanding of individual phenotypes, enabling the design of truly personalized neurogenomics-based functional plans. This article outlines the steps of the proposed integrative neurogenomics workflow, discussing its advantages and limitations, and presents highlights from an illustrative case intended as a potential reference model to establish the foundation for a new standard of personalized genomic medicine in neurodegeneration. The workflow underscores the importance of considering the additive burden of genetic variants typically classified as benign—beyond the ACMG pathogenicity framework—for accurate phenotypic assessment. It further demonstrates the feasibility of developing actionable and highly precise functional interventions by integrating genomic and biochemical data. Findings from the case example reveal correlations between genetic variants and biochemical markers, providing the basis for personalized recommendations in nutrition, lifestyle, and supplementation. This framework aims to establish the foundations of personalized genomic medicine in neurodegenerative diseases, underscoring the urgent need to move beyond one-size-fits-all approaches.

## 1 Introduction

Despite decades of intensive research and substantial investment—with Alzheimer’s disease alone projected to surpass 1 trillion USD in global cost by 2050 (Nandi et al., 2024), therapeutic progress in neurodegenerative disorders has remained limited, with most clinical approaches continuing to target downstream proteinopathies and yielding only modest effects on disease trajectories (Selkoe and Hardy, 2016; Bloom, 2014; Wu and Fuh, 2025; Anderson et al., 2017; Gauthier et al., 2016; Waite, 2015; Klein, 2002). Pharmaceutical companies have invested hundreds of millions of dollars in developing disease-modifying drugs, including monoclonal antibodies, inhibitors (e.g., aducanumab, donanemab, donepezil, memantine), while neurologists, psychiatrists, foundations, families, and affected individuals continue to await a transformative therapeutic breakthrough (Knopman et al., 2021).

Functional protocols emphasize the importance of evaluating infectious, inflammatory, metabolic, hormonal, toxic, and genetic variants, and have shown modest success in preventing, and in some cases partially reversing, cognitive decline, as reported in a pilot study published in the Journal of Alzheimer’s Disease (Toups et al., 2022). However, their effectiveness requires extreme adherence, and important gaps remain, particularly in the genetic dimension.

Initially limited to APOE genotyping, neurogenomic clinical recommendations have evolved toward broader SNP panels, covering several biological pathways relevant to neurodegeneration. Nevertheless, these panels still provide a limited genomic perspective, often lacking the scope and precision required for individualized interpretation (Villalba et al., 2023).

Through the lens of genomics and molecular biology there is a great complexity inherent to cellular processes and their interactions, not to mention the pitfalls in variant filtering and classification, including potential errors in alignment, annotation, and variant calling, as well as the challenges of pathogenicity classification under ACMG/AMP guidelines (Richards et al., 2015). Moreover, there is still no universally accepted framework for clinical variant filtering and classification across medical genetics— different platforms, different results, different interpretation. Crucially, most neurodegenerative phenotypes—particularly late-onset forms—are polygenic and multifactorial, frequently arising from variants of incomplete penetrance and variable expressivity. Consequently, most genetic contributions to neurodegenerative phenotypes escape the strict pathogenic/benign dichotomy defined by ACMG, creating a bioinformatic challenge: common variants of small effect—and even variants classified as benign—may exert additive influences on phenotype. While polygenic risk scores (PRS) address part of this architecture by aggregating validated risk alleles from GWAS, the proposed workflow further considers the potential cumulative burden of variants typically deemed benign, aiming to bridge the gap between population-level risk estimation and individualized interpretation (Torkamani et al., 2018).

Compounding these challenges is the historical limited integration of genetics, genomics, and precision medicine into medical, neurological and psychiatric training—often no more than a brief overview of classical genetics. Given that neurogenomics, oncogenomics, cardiogenomics, among the others genomic-related medical specialties have only emerged as dedicated fields within the past two decades, there is a pressing need for ongoing medical education and for the integration of advanced genomics into residency and specialty curricula (Plunkett-Rondeau et al., 2015).

Recognizing these limitations, clinical practice has increasingly shifted away from restricted SNP panels toward what is widely considered one of the most cost-effective strategies to date: whole-exome sequencing (WES; Goh and Choi, 2012; Rabbani et al., 2014), including mitochondrial genome (mtDNA) analysis. Today, the cost of WES is comparable to what SNP-based panels required years ago. However, WES enables the comprehensive analysis of approximate ~19,500 protein-coding genes and, depending on the library (e.g., Agilent SureSelect CRE V4), approximately 5,500 additional clinically relevant loci, including splice sites, untranslated regions, lncRNAs, pseudogenes, and regulatory elements. Structural CNVs ranging from ~1 kb to several megabases may also be inferred from coverage depth, although this typically requires complementary bioinformatic analysis or additional assays (Petersen et al., 2017; Need et al., 2012; Corominas et al., 2022; Bertier et al., 2016).

### 1.1 Diagnostic challenges and the need for an integrative genomic framework

Neurodegenerative disorders—including Alzheimer’s disease (AD), Parkinson’s disease, frontotemporal dementia (FTD), dementia with Lewy bodies (DLB), amyotrophic lateral sclerosis (ALS), progressive supranuclear palsy (PSP), among others—represent one of the most pressing challenges for modern medicine. These conditions are often multifactorial and heterogeneous, except for the rare cases caused by fully penetrant variants (Hardy and Escott-Price, 2025; Dilliott et al., 2021; Pihlstrøm et al., 2018; Cocoș and Popescu, 2024). Their clinical manifestations frequently overlap with psychiatric syndromes, delaying recognition (Menculini et al., 2021; Tsoukra et al., 2022; Maristany et al., 2024), and when finally diagnosed, patients are often labeled with phrases such as “there is no cure” or “we will try to slow progression,” while standard protocols focus primarily on symptomatic pharmacological management of proteinopathies (e.g., amyloidosis, tauopathies, prionopathies, synucleinopathies), without addressing their molecular origins (Scheltens et al., 2021).

A central obstacle lies in their complex etiology: most cases result from polygenic contributions of multiple variants classified as benign, in interaction with multifactorial exposures across the lifespan. While monogenic forms exist—typically rare, early-onset, and associated with high penetrance—late-onset familial cases often display incomplete penetrance, with carriers remaining asymptomatic until midlife or after hormonal transitions such as menopause and andropause (Breeze et al., 2024; Henderson, 2014; Mervosh and Devi, 2025; Jansen et al., 2019). Conversely, individuals without family history may develop cognitive decline due to cumulative exposomic influences (Monaco et al., 2025).

Conventional diagnostic pathways remain largely anchored in clinical guidelines, neuroimaging, and basic cognitive tests such as the Montreal Cognitive Assesment (MoCA), the Mini-Mental State Examination (MMSE) or disease-specific questionnaires like the Parkinson’s Disease Questionnaire-39 (PDQ-39) (Folstein et al., 1975; Porsteinsson et al., 2021; Gadhave et al., 2024). While structural and functional neuroimaging can localize damage, they cannot explain its molecular origin (Huang and Zhang, 2023; Song et al., 2025). Similarly, cerebrospinal fluid (CSF) biomarkers provide insight into neuronal injury but, rather than contextualizing the underlying genomic contributions, they may only aid gene discovery (Neumann et al., 2023; Omar and Preddy, 2020). Consequently, patients are frequently diagnosed only at advanced stages and are often referred directly to palliative care (Taylor et al., 2019).

In genetic diagnostics, reliance on commercial gene panels further complicate the problem. These panels are curated to target a limited set of genes, leaving hundreds of thousands of potentially relevant variants unexplored. As a result, false negatives and false positives are frequent. Moreover, following ACMG guidelines (Richards et al., 2015) a large proportion of variants can be classified as of uncertain significance (VUS) or benign, without considering their additive burden in shaping polygenic phenotypes. This leaves a significant blind spot in clinical reporting and fails to capture the subtle but cumulative influence of common variants.

Recent advances, including genome-wide association studies (GWAS), have identified hundreds of loci associated with neurodegenerative risk. These findings underscore that not only rare pathogenic mutations, but also common variants exert modifying and additive effects on disease trajectories (Chung et al., 2023; Andrews et al., 2023). Still, the integration of such knowledge into clinical workflows remains limited. The result is a diagnostic system that, despite rapid advances in imaging, fluid biomarkers, and omics sciences, often provides only partial answers.

Taken together, these limitations highlight the urgent need for updated frameworks that transcend reductionist approaches and integrate genomic, metagenomic, biochemical, nutritional, and lifestyle dimensions into a comprehensive precision-medicine strategy for neurodegeneration.

In this manuscript, we propose that an integrative neurogenomics workflow—combining multi-omic phenotype–oriented whole-exome sequencing, biologically-oriented common variant burden profiling, biochemical markers, functional metagenomic data, and multidisciplinary clinical interpretation—can generate testable, personalized diagnostic and therapeutic hypotheses in neurodegenerative disorders. This workflow serves as a falsifiable conceptual model—already implemented in clinical practice—that can be prospectively evaluated in future studies.

To contextualize the need for this integrative approach, Table 1 provides a concise comparison including benefits and drawbacks between existing diagnostic and therapeutic frameworks, and the added mechanistic value offered by the proposed workflow (De Strooper and Karran, 2016; Guerreiro and Hardy, 2014; Hampel et al., 2018; Richards et al., 2015; Torkamani et al., 2018; Torkamani et al., 2018; Chatterjee et al., 2016; Lambert et al., 2023; Green et al., 2013; Lee K. et al., 2025; Califf, 2018; FDA-NIH Biomarker Working Group, 2016; Gilbert et al., 2018; Proctor et al., 2019; Quince et al., 2017; Ndoro, 2014; Taberna et al., 2020).

**Table 1:** Summary table comparing diagnostic and therapeutic approaches used in neurodegenerative disorders and their relationship to the proposed integrative neurogenomics workflow.

Framework/Domain	Type of approach	Core features	Benefits	Drawbacks/limitations
AT(N) Framework	Diagnostic-only	Classifies Alzheimer’s disease based on amyloid (A), tau (T), and neurodegeneration (N) biomarkers	Anchored in widely used biological markers; standardized research classification	Lacks genomic and metabolic context; etiology-agnostic; limited mechanistic insight; not individualized
Structural/Functional Neuroimaging	Diagnostic-only	MRI, CT, FDG-PET, SPECT to localize atrophy, hypometabolism, and network dysfunction	Essential for differential diagnosis; reveals anatomical and functional patterns	Lacks genomic context; detects downstream effects, not molecular causes; limited temporal sensitivity; high cost
CSF/Plasma Neurodegeneration Biomarkers	Diagnostic-only	Measures amyloid, tau, NfL, synaptic markers	Provides biochemical evidence of neuronal injury; correlates with disease stage	Lacks genomic and metabolic context; limited specificity for upstream drivers; invasive (CSF)
Targeted Gene Panels	Diagnostic-only	Tests limited sets of genes associated with specific neurodegenerative syndromes	Useful for monogenic/early-onset forms; rapid results	High false-negative and-positive interpretation; excludes other relevant loci; does not assess polygenic burden; lacks pathway aggregation; narrow genomic scope
Pharmacological Therapy	Therapeutic-only	Cholinesterase inhibitors, NMDA antagonists, anti-amyloid mAbs	Standard of care; symptomatic relief; structured evidence-based clinical guidelines	Limited impact on disease progression; not personalized; effectiveness varies by genotype, metabolism, and molecular context
Multidomain Lifestyle/Functional Protocols	Diagnostic + Therapeutic	Addresses metabolic, inflammatory, vascular, hormonal and lifestyle pathways	Holistic, multi-system approach; early evidence of benefit in mild cognitive impairment	Requires strict adherence; limited genomic personalization; variable evidence base; not mechanistically individualized
Integrative Neurogenomics Workflow (proposed model)	Diagnostic + Therapeutic	Multi-omic integration (WES, PRS-F, biochemical markers, metagenomic pathways) interpreted through a multidisciplinary team to generate mechanistic, personalized interventions	Adds etiological, genomic, metabolic, and microbial mechanistic layers; links genotype - pathway - phenotype - intervention; complements standard frameworks; enables personalized prevention and treatment	Requires strict adherence, sequencing, bioinformatics and MDT coordination; currently limited by cost, accessibility, and need for specialist training

### 1.2 Stepwise integrative neurogenomics workflow

This workflow is primarily designed for Alzheimer’s disease, frontotemporal dementia, dementia with Lewy bodies, Parkinson’s disease, ALS and PSP. It is intended for neurologists, psychiatrists, clinical geneticists and precision-medicine practitioners seeking to incorporate multi-omic interpretation into neurodegenerative care.

Operationally, the workflow follows a stepwise sequence: clinical phenotype evaluation precedes whole-exome sequencing, as the former defines the diagnostic axes required for phenotype-directed variant analysis. After this initial phase, the remaining components—WES, biochemical markers, and metagenomic profiling—may be obtained either sequentially or in parallel depending on clinical urgency, logistics, and available resources.

Their interpretation, however, is inherently integrative and iterative: each dataset—genomic variants, biochemical abnormalities, polygenic modulation, and microbiome-derived pathways—refines the others as new information becomes available, allowing the multidisciplinary team to recalibrate the emerging clinical model. Similarly, the functional precision plan may begin with early biochemical findings and subsequently be adjusted as genomic and metagenomic data are incorporated.

The following sections outline the operation sequence of the workflow and the integrative interpretation that emerges from it.

### 1.3 Phenotype-based clinical evaluation

The initial clinical evaluation is conducted by the medical coordination team (general practitioner and head nurse) through structured anamnesis. All available neuroimaging studies and conventional laboratory tests are requested to integrate medical history. Subsequently, during the first consultation, the clinical geneticist begins to assess the extent of genetic contribution to the patient’s condition.

In dementias, particularly Alzheimer’s disease (AD), fewer than 5% of cases correspond to a purely genetic etiology, usually suspected in patients with a clear autosomal dominant family history or early-onset disease (<65 years). For these cases, *PSEN1, PSEN2,* and *APP* are considered mandatory genes for analysis (Ibanez et al., 2021; Zampatti et al., 2021). In Parkinson’s disease (PD), genetic testing is recommended regardless of whether the presumed etiology is monogenic or multifactorial, especially in patients with onset before the age of 50 (Di Fonzo et al., 2018; Pal et al., 2023). For amyotrophic lateral sclerosis (ALS), current guidelines recommend at least molecular analysis of *C9ORF72, SOD1, FUS,* and *TARDBP* (Roggenbuck et al., 2023). When the clinical presentation is consistent with a disorder that is unequivocally genetic in origin, such as Huntington’s disease, requesting the specific molecular assay (repeat expansion testing) remains the most appropriate approach. We do not include further phenotypic examples here in order to avoid excessive length.

As part of our process, we established a Multidisciplinary Team (MDT) given the substantial advantages of interdisciplinary dialogue (Ndoro, 2014; Taberna et al., 2020). In this initial stage, the MDT is composed of the head nurse, the general practitioner, a neurologist—all instructed or at least familiarized with neurogenomics—the clinical geneticist, and the precision medicine and clinical genomics specialist. Together, they outline a personalized pathway for the patient and determine the required analyses. Established dementia diagnostic frameworks—including AT(N) and fluid biomarkers integration—are incorporated into the multidisciplinary evaluation whenever available. Unless otherwise required, for the pathologies described above we request Whole Exome Sequencing (WES) with phenotype-directed analysis, a selected panel of blood-based biochemical markers, and—where financially feasible—the functional metagenomic analysis of the gut microbiome, which provides significant added value (Gilbert et al., 2018; Proctor et al., 2019; Quince et al., 2017).

### 1.4 Whole exome sequencing (WES): justification and interpretation

#### 1.4.1 Advantages over panels

As mentioned in the introduction, WES enables the comprehensive analysis of ~19,500 protein-coding genes and, depending on the library (e.g., Agilent SureSelect CRE V4), approximately 5,500 additional clinically relevant loci, including splice sites, untranslated regions, lncRNAs, pseudogenes, regulatory elements and mtDNA (Petersen et al., 2017; Need et al., 2012; Corominas et al., 2022; Bertier et al., 2016). These characteristics make Whole Exome Sequencing (WES), remain, to date, one of the most cost-effective options for comprehensive genetic diagnosis in neurodegenerative disorders. Gene panels inevitably exclude variants in genes that may be relevant for a given phenotype, whereas WES provides the ability to analyze additional variants in coding regions as required. Moreover, WES allows for the integration of pharmacogenomic information, particularly exonic variants in genes related to the Absorption, Distribution, Metabolism, and Excretion (ADME) of drugs, which may complement targeted assays traditionally used to calculate pharmacogenetic profiles, with the possibility of expanding the understanding of gene-drug interactions, and indirectly inform potential drug–drug and drug-nutrient interactions—which will necessarily have to be calculated through other platforms (Whirl-Carrillo et al., 2012). In addition, as the Variant Call Format (VCF) file of every patient is already generated, if future scientific literature highlights new variants, or genes of clinical relevance, there is no need to obtain a new sample or repeat an expensive sequencing procedure; the data can simply be re-analyzed bioinformatically. Furthermore, if another pathology emerges in the future for the same patient—for example, a tumor—the MDT, expanded to include an oncologist and a pathologist—can rapidly analyze genes associated with hereditary cancer, using the same dataset. WES can be considered a powerful genetic tool with great potential to address diverse phenotypes as they arise, with an efficient turnaround time at the request of both, patients and physicians.

#### 1.4.2 Secondary and incidental findings

Another major advantage of WES is the possibility to obtain secondary and incidental findings prior informed consent of the patient. According to the policy statement of the ACMG on clinical sequencing (Green et al., 2013), it is of utmost importance to alert the patient to the possibility of such results in pretest patient discussions, clinical testing, and reporting of results—secondary findings list most recently updated to version 3.3 (Lee K. et al., 2025). These insights are of great value in preventing unanticipated clinical risks. As an example, during the evaluation of a patient whose primary phenotype was attention-deficit/hyperactivity disorder (ADHD), an incidental finding revealed a pathogenic nonsense mutation in *SCN5A*, associated with Brugada syndrome. Since the patient was an extreme athlete, this finding with its respective recommendation, may have prevented a potentially fatal episode of sudden cardiac death.

#### 1.4.3 Limitations of benign and VUS classifications

Ultimately, WES is not a genetic oracle, and in contrast to Mendelian diseases– which often result from single-gene coding variants (Botstein and Risch, 2003), several complex traits could be driven by noncoding variants that presumably affect gene regulation. There are complex scenarios in which WES must be expanded to whole-genome sequencing (WGS)—thus engaging with the remaining 98.5% of the genome—when intronic regions of potential clinical relevance are suspected—for instance, non-exonic pathogenic variants (Hiraide et al., 2022). Likewise, there are genetic alterations for which WES is not the appropriate technique, such as indels larger than 50 base pairs, tandem repeat expansions, structural rearrangements (inversions, translocations), or variants located in highly repetitive or low-coverage regions. Additional omics layers, including epigenomics and transcriptomics, may also be required. Despite these limitations, WES continues to offer one of the most favorable cost–benefit outcomes, offering almost the same percentage of diagnostic yield as Whole Genome Sequencing (WGS), but with and substantially lower bioinformatics workload (Nurchis et al., 2023).

#### 1.4.4 MDT review of variant prioritization

For the preparation of the genetic results report, the workflow relies again on the MDT, which at this step includes at least two clinical geneticists, a bioinformatician, a molecular biologist, a pharmacogenomics specialist, and a precision medicine expert. Together, this team determines which variants should be reported, how they should be classified, and what recommendations should be made (Table 2).

**Table 2:** Genomic DATA

Reporting category	Gene/variant (rsID)	Mechanistic domain(s)	Biochemical/phenotype relevance in this case
Linked to clinical phenotype	*ATP7B* – rs121907998	Copper transport, metal homeostasis	Supports monitoring of ceruloplasmin/free copper; aligns with oxidative stress vulnerability.
Linked to clinical phenotype	*HFE* – rs1799945	Iron metabolism, redox regulation	Consistent with oxidative stress pathways; relevant to homocysteine and vascular markers.
Linked to clinical phenotype	*LRP8* – rs5174	LDL receptor signaling, vascular risk	Supports elevated atherogenic index; vascular contribution to cognitive decline.
Linked to clinical phenotype	*CAPN10* – rs3792267	Insulin signaling, glucose metabolism	Consistent with hyperinsulinemia; supports metabolic component of phenotype.
Secondary finding (ACMG v3.3)	*BTD* – rs13078881	Biotin recycling, cofactor metabolism	Partial enzyme reduction; relevant to micronutrient interpretation and supplementation decisions.
Incidental finding	*FCN3* – rs4494157	Complement activation, innate immunity	Suggests immune/inflammatory modulation; aligns with inflammatory markers.
Incidental finding	*NCR3* – rs11575837	NK-cell activation, innate immunity	Potential influence on immune tone and neuroinflammatory susceptibility.
Incidental finding	*CHI3L1* – rs4950928	Microglial activation, neuroinflammation	Supports inflammatory pathway involvement; relevant to YKL-40 axis.
Incidental finding	*GABRA2* – rs279871	GABAergic neurotransmission	Modulates anxiety/behavioral regulation; contextualizes neuropsychiatric profile.
Incidental finding	*NPSR1* – rs324981	Stress/arousal circuits, neuroimmune signaling	May influence anxiety, arousal, inflammatory responses; supports clinical phenotype.
Incidental finding	*HLA*-A – rs1061235	Drug hypersensitivity, immune response	Relevant for pharmacovigilance (SCAR risk) in future prescribing.
Incidental finding	*LOXL1* – rs1048661	Elastogenesis, ECM integrity	Associated with exfoliation syndrome; relevant to ophthalmologic evaluation.

References
Merico et al. (2020), Zhang et al. (2020)
Liu et al. (2011)
Yaralı et al. (2024)
Onyango et al. (2023)
Gomaa et al. (2021)
Shen et al. (2012), Shen et al. (2014)
Connolly et al. (2023), Ohi et al. (2010)
Ezzidi et al. (2010)
Sakai et al. (2010)
McCormack et al. (2011)
Lennertz et al. (2012), D’Amato et al. (2010)
Lemmelä et al. (2009), Ozaki et al. (2008)

### 1.5 Polygenic risk scores (PRS)

As discussed in previous sections, exome sequencing provides a deeper understanding of the genetic basis of complex disorders, since it enables a global analysis not only of pathogenic variants but also of risk variants, variants of uncertain significance (VUS), and benign variants. This has contributed to addressing the question of “missing heritability”—while rare pathogenic variants explain a portion of a trait, adding the modest impact of common variants helps account for the unexplained heritability observed in many conditions. It is therefore not surprising that, based on the premise that the cumulative effect of subtle variations in key genes or regulatory pathways contributes to disease vulnerability (Boyle et al., 2017), risk must be estimated through a polygenic risk score. This approach provides a risk estimate derived from the aggregate effect of validated common risk alleles identified through GWAS, reflecting how multiple variants of modest effect jointly contribute to the polygenic nature of the disease. Polygenic risk scoring is thus a powerful tool for risk stratification (Escott-Price et al., 2015) and, when integrated with other biomarkers, an emerging component of precision medicine.

It is important to clarify that, whereas population-level PRS models require formal effect-size modeling, linkage disequilibrium correction, ancestry calibration, and validation across large cohorts, the score used in this workflow is a functional polygenic score (PRS-F) designed for mechanistic, individual-level interpretation rather than for epidemiological prediction.

Since polygenic risk score calculation requires large GWAS datasets, some neurodegenerative disorders such as frontotemporal dementia (FTD) or ALS currently lack well-established standards like those available for AD or PD (Lambert et al., 2023). Nevertheless, preliminary estimates can be derived based on the studies currently available.

The involvement of the MDT is also essential for an accurate evaluation of the PRS. This begins with assessing the methodology used, given the variability across approaches. The clinical geneticist and bioinformatician ensure that the calculation is accurate and reproducible; the specialist confirms that the score aligns with the patient’s phenotype; and the precision medicine and clinical genetics experts contextualize the PRS alongside other biomarkers, analyzing genetic predisposition in relation to the epigenetic burden of lifestyle, diet, and supplementation factors (Table 3).

**Table 3:** Functional polygenic risk score.

Gene/variant	Evidence	Risk/protection	Estimated weight
*GRN* rs5848	Associated with reduced progranulin and risk of FTLD	Very High Risk	High
*CHI3L1* rs4950928	Associated with elevated YKL-40 and Alzheimer’s disease	High Risk	Medium–High
*HFE* rs1799945	Iron overload, increased oxidative stress	High Risk	Medium
*ATP7B* rs121907998	Wilson carrier, mild neurological symptoms	High Risk	Medium
*ABCA7* rs3752246; rs4147929; rs3764650	Late-onset Alzheimer’s risk, haplotype dependent	High Risk	Low–Medium
*LRP8* rs5174	Cardiovascular risk and vascular cognitive decline	High Risk	Low–Medium
*APOE* rs440446	Benign, minor effect on expression	Neutral/Risk	Low
*NPSR1* rs324981	Psychiatric risk, verbal memory	High Risk	Low
*LRRK2* rs7133914	Protective factor against Parkinson’s disease	Protection	Moderate

*Estimated polygenic score:* (1) Major risk factors: *GRN, CHI3L1*. (2) Moderate risk factors: *HFE, ATP7B, ABCA7, LRP8*. (3) Minor risk factors: *APOE* promoter*, NPSR1*. (4) Protective factor: *LRRK2* Arg1398His. *Integrated result:* The weighted polygenic score places the patient at a moderate-to-high risk (percentile ~80–85%) for neurocognitive disorders, with greater susceptibility to frontotemporal lobar degeneration and late-onset Alzheimer’s disease.

### 1.6 Biochemical and metabolic profiling

The pathogenesis of neurodegenerative disorders generally involves a complex physiopathology that includes age-related changes, abnormal function and accumulation of brain proteins, inflammation, disruption of energy homeostasis, hormonal imbalances, metabolic imbalances, DNA damage, among other processes (Gadhave et al., 2024). These mechanisms show signs of occurrence many years before the clinical symptoms of the phenotype become apparent. Therefore, the targeted identification and management of key metabolic, infectious, inflammatory, hormonal, and toxic biomarkers (Table 4) is essential to obtain a clear picture of the specific dysfunctions and alterations present in each individual, with the aim of restoring homeostasis and addressing the “aggressors” that may represent the true drivers of the underlying pathophysiology.

**Table 4:** Biochemical markers.

**Complete blood count (CBC)**				
**Red blood cell parameters**				
**Parameter**	**Result**	**Unit**	**Reference range**	**Status**
Erythrocytes	4.75	10^6^/μL	4.50–6.00	Normal
Hemoglobin	15.9	g/dL	14.2–17.8	Normal
Hematocrit	45.1	%	43.00–53.00	Normal
Mean Corpuscular Volume (MCV)	94.9	fL	83.00–98.00	Normal
Mean Corpuscular Hemoglobin (MCH)	33.6	pg	28.00–34.00	Normal
Mean Corpuscular Hemoglobin Concentration (MCHC)	35.3	g/dL	32.00–35.00	Borderline
Red Cell Distribution Width (RDW)	13	%	11.60–14.60	Normal

**Platelet parameters**				
**Parameter**	**Result**	**Unit**	**Reference range**	**Status**
Platelets	207	10^3^/μL	200–400	Normal
Mean Platelet Volume (MPV)	8.2	fL	8.20–12.20	Normal

**White blood cell parameters**				
**Parameter**	**Result**	**Unit**	**Reference range**	**Status**
Leukocytes	7	10^3^/μL	6–10	Normal
Neutrophils (%)	69.1	%	50.00–70.00	Normal
Lymphocytes (%)	23.5	%	20.00–40.00	Normal
Monocytes (%)	5.9	%	4.00–12.00	Normal
Eosinophils (%)	1.1	%	1.00–5.00	Normal
Basophils (%)	0.4	%	0.00–1.00	Normal
Absolute Neutrophils	4.8	10^3^/μL	2.5–7.0	Normal
Absolute Lymphocytes	1.7	10^3^/μL	1.0–4.0	Normal
Absolute Monocytes	0.4	10^3^/μL	0.2–1.2	Normal
Absolute Eosinophils	0.1	10^3^/μL	0.0–0.5	Normal
Absolute Basophils	0	10^3^/μL	0.0–0.2	Normal

Biochemistry				
Glycated hemoglobin				
Parameter	Result	Unit	Reference range	Status
HbA1c	4.9	%	<5.3	Optimal

Comprehensive metabolic panel (CMP, 30 elements)				
**Parameter**	**Result**	**Unit**	**Reference range**	**Status**
Glucose	88	mg/dL	70–90	Normal
Blood Urea Nitrogen (BUN)	15.23	mg/dL	7.00–25.00	Normal
Urea	33	mg/dL	15–54	Normal
Creatinine	1.01	mg/dL	<1.2 (men)	Normal
Uric Acid	6.8	mg/dL	4.4–7.6	Normal
Sodium	141	mmol/L	135–145	Normal
Potassium	4.03	mmol/L	4.5–5.5	Low
Chloride	106	mmol/L	98–111	Normal
Calcium (total)	9.6	mg/dL	8.5–10.5	Normal
Phosphorus	3.13	mg/dL	2.40–4.70	Normal

Lipid profile				
Parameter	Result	Unit	Reference range	Status
Total Cholesterol	189	mg/dL	150–200	Normal
HDL Cholesterol	35	mg/dL	>50	Low
Triglycerides	108	mg/dL	40–90	Elevated
LDL Cholesterol	132.48	mg/dL	<100	Elevated
VLDL Cholesterol	22	mg/dL	<20 (Desirable)	Elevated
Atherogenic Index	5.4	–	<5.0 (men)	Elevated

Proteins				
Parameter	Result	Unit	Reference range	Status
Total Proteins	6.71	g/dL	6.10–7.90	Normal
Albumin	4.58	g/dL	4.5–5.5	Normal
Globulins	2.13	g/dL	1.90–4.00	Normal
Albumin/Globulin Ratio	2.15	–	1.10–2.30	Normal

Liver function				
Parameter	Result	Unit	Reference range	Status
Total Bilirubin	0.87	mg/dL	0.30–1.00	Normal
Direct Bilirubin	0.15	mg/dL	0.03–0.18	Normal
Indirect Bilirubin	0.72	mg/dL	0.27–0.82	Normal
AST (SGOT)	23	U/L	<25	Normal
ALT (SGPT)	27	U/L	<25	Slightly Elevated
LDH	142	U/L	98–192	Normal
Alkaline Phosphatase	45	U/L	34–104	Normal
Gamma-Glutamyl Transferase (GGT)	11	U/L	9–64	Normal

Other parameters				
Parameter	Result	Unit	Reference range	Status
Creatine Kinase (CK, Total)	45	U/L	30–223	Normal
Iron	128	μg/dL	50–212	Normal

Immunochemistry				
Vitamins				
Parameter	Result	Unit	Reference range	Status
Serum Folate	18	ng/mL	11–25	Normal
Vitamin B12	5,277	pg/mL	500–1,500	Very Elevated

Hormones				
Parameter	Result	Unit	Reference range	Status
Cortisol (AM)	10.47	μg/dL	10–18	Normal
Insulin	8.54	μIU/mL	3–5.5	Elevated
TSH	2.651	μIU/mL	<2	Elevated
Free T3	2.53	pg/mL	3.2–4.2	Low
Free T4	0.92	ng/dL	1.3–1.8	Low

Automated serology				
Parameter	Result	Unit	Reference range	Status
Progesterone (Pg)	0.2	ng/mL	≤0.2	Normal
FSH	2.84	mIU/mL	0.95–11.95	Normal
hs-CRP (Cardiac)	0.41	mg/L	Low risk <1	Normal
Total Testosterone	321.53	ng/dL	500–1,000 (men)	Low
Estradiol (E2)	<20.00	pg/mL	<39 (men)	Normal
LH	1.3	mIU/mL	0.57–12.07	Normal
Prolactin	7.93	ng/mL	3.46–19.40	Normal

Specialized tests				
Vitamin D				
Parameter	Result	Unit	Reference range	Status
Vitamin D (25-OH, Total)	45.4	ng/mL	50–80	Low
Vitamin D3 (25-OH)	45.4	ng/mL	–	Normal
Vitamin D2 (25-OH)	<1.0	ng/mL	–	Normal

Steroid hormones				
Parameter	Result	Unit	Reference range	Status
Pregnenolone	21	ng/dL	100–250	Low
DHEA-Sulfate	55	μg/dL	150–500 (men)	Low

Heavy metals				
Parameter	Result	Unit	Reference range	Status
Blood Cadmium	<0.5	μg/L	<2.5	Normal
Blood Arsenic	<3	μg/L	<7,000 ng/mL	Normal
Blood Lead	2.8	μg/dL	<2	Slightly Elevated
Blood Mercury	<5	μg/L	<4	Slightly Elevated

Cardiovascular marker				
Parameter	Result	Unit	Reference range	Status
Homocysteine	8.1	μmol/L	4–7	Elevated

Among the clear clinical benefits are the improvement of differential diagnosis, stratification of prognosis, and monitoring of disease progression, as well as the guidance of interventions targeting modifiable factors (Alcolea et al., 2023; Qu et al., 2023; McKeever et al., 2025).

Once again, the MDT provides substantial value at this stage, working interdisciplinarily to determine which metabolic axes exert the greatest influence on the clinical phenotype, whether these are linked to the genetic variants identified, and the most appropriate approach to address them—whether pharmacological, or through supplementation, nutrition, and lifestyle interventions. Precise decision-making is indispensable both to avoid iatrogenesis and to optimize outcomes. This step also initiates the multidisciplinary meeting described in the following section, where all data streams are integrated.

### 1.7 Multidisciplinary team consolidation

At this stage, the bioinformatics team integrates all available data (genomic, polygenic, biochemical, and, when applicable, metagenomic) integrates all available data (genomic, polygenic, biochemical, and, when applicable, metagenomic) (Tables 2–4) into a single consolidated report (Table 5), highlighting abnormal values and enabling a clear overview to better identify therapeutic windows. In this final interdisciplinary diagnostic meeting, all specialists contribute their perspectives in order to reach a consensus on both diagnosis and treatment. For cases in which cell and gene therapies may represent promising options, the corresponding specialist is also invited to the committee.

**Table 5:** Personalized functional plan.

Personalized functional recommendations		
Gene	Finding	Functional recommendation
*ATP7B (Heterozygous, pathogenic)*	Mild copper accumulation	Supplement with chelated zinc 25–50 mg/day (antagonizes copper); avoid copper-rich foods (liver, shellfish, cocoa); monitor free copper and ceruloplasmin (Leuci et al., 2025).
*HFE (H63D)*	Risk of mitochondrial dysfunction and neurodegeneration due to iron	Avoid supplemental iron; assess ferritin and transferrin; promote periodic blood donations if overload is present (Lehmann et al., 2012; Yu et al., 2025).
*BTD* (Heterozygous)	Partial biotinidase deficiency	Biotin 5–10 mg/day as essential coenzyme for neuronal metabolism (Almasi et al., 2024).
*LRP8, FCN3, CHI3L1, GABRA2, NPSR1, CAPN10*	Mild neurovascular, anxious, or immunological dysfunction	Natural anti-inflammatories (curcumin, EGCG, omega-3); aerobic exercise + neurofeedback; mitochondrial support: CoQ10 + PQQ + NMN taken with TMG or methylfolate (below; Francis et al., 2024; Shi et al., 2022; Feng et al., 2016; Ebrahimi et al., 2023).
*CHI3L1* (Homozygous)	High neuroinflammatory risk	Priority: reduce oxidized glutathione and CRP; NAC 600 mg q12h, ALA 300 mg/day, Resveratrol 250–500 mg/day (Pei et al., 2024).
Objective: Optimize hormones, reduce toxicity, and improve mitochondrial, vascular, and immune function.		

Metabolism and biochemistry	
Alteration	Recommended intervention
Subclinical hypothyroidism (high TSH, low T3/T4)	Support with tyrosine (500–1,000 mg fasting) + selenium (200 mcg) + zinc + ashwagandha; consider bioidentical T3/T4 therapy if symptoms persist (Mooradian and Haas, 2025; Duță et al., 2025; Speers et al., 2021; Guo and Rezaei, 2024).
Elevated insulin (8.54)	Intermittent fasting 16:8; Berberine 500 mg q12h or Metformin or Berberine; eliminate simple carbs and starches (Elias et al., 2023; Lv et al., 2025; Gasmi et al., 2024).
Hormonal deficiency (testosterone, DHEA, pregnenolone)	Bioidentical Pregnenolone 30–50 mg/day (sublingual or micronized); Bioidentical DHEA 25 mg/day; testosterone gel or IM if clinically required (Osmanovic-Barilar and Salkovic-Petrisi, 2016; Lee B. H. et al., 2025).
Moderate low vitamin D (45.4)	Supplement with Vitamin D3 1,000 IU/day + K2 (MK7; Wang et al., 2023; Mirarchi et al., 2023).
Elevated homocysteine (8.1)	Methylfolate 800 mcg + B6 (25 mg), add TMG 500–1,000 mg/day if not reduced (Hara et al., 2016; Pi et al., 2020).
Dyslipidemia (low HDL, high triglycerides, elevated LDL)	Omega-3 (EPA/DHA) at least 2,000 mg/day (krill phospholipids); niacin 50 mg/day, soluble fiber (psyllium, seeds); cardiovascular exercise (Chitre et al., 2020).
Lead & mercury toxicity	Chlorella + cilantro + liposomal glutathione 500–1,000 mg/day; consider EDTA or DMSA under clinical supervision (Iskusnykh et al., 2022).

Weekly nutrition plan			
Day	Breakfast (10:00)	Lunch (14:00)	Light Dinner (17:30)
Monday	Green tea + spinach omelet with turmeric & avocado	Baked salmon with asparagus & quinoa	Bone broth + nuts
Tuesday	Black coffee + chia in coconut milk with berries	Organic chicken with kale salad, walnuts & EVOO dressing	Miso soup + ½ avocado
Wednesday	Green smoothie (cucumber, celery, ginger, turmeric, spirulina) + 2 boiled eggs	Lettuce tacos with lean meat, guacamole & pico de gallo	Cauliflower purée + sardines
Thursday	Matcha tea + avocado with hemp seeds & lemon	Steamed white fish with broccoli & turmeric	Lentil soup + green salad
Friday	Coconut yogurt + nuts + turmeric	Salmon bowl with cauliflower rice, avocado & sprouts	Pumpkin & ginger cream soup
Saturday	Green juice + poached eggs with mushrooms	Warm tuna salad with spinach, beets & walnuts	Chicken soup with turmeric & ginger
Sunday	Coffee with MCT oil + almonds	Grilled white meats with sweet potato purée & watercress salad	Rooibos tea + 1 boiled egg

*General guidelines:* (1) Intermittent fasting 16:8 (last meal at 18:00 h). (2) Hydration: 2.5–3 L water/day + natural electrolytes. (3) Avoid: sugar, dairy, refined flours, cereals, refined vegetable oils. (4) Prioritize: vegetables, omega-3, healthy fats, clean protein.

A particularly important component, to which we devote significant emphasis, is the ethical dimension—especially the communication of results. This becomes critical in the presence of pathogenic variants of any type (phenotype-related, secondary, or incidental), where therapeutic options may be limited or absent. In these scenarios, we aim to ensure clarity of information (avoiding false expectations) while maintaining humanity and compassion in the delivery of results, with the goal of minimizing any potential for anxiety, depression, panic, or psychological distress (Weiner, 2014).

### 1.8 Construction of a functional precision plan

**Figure 1:**
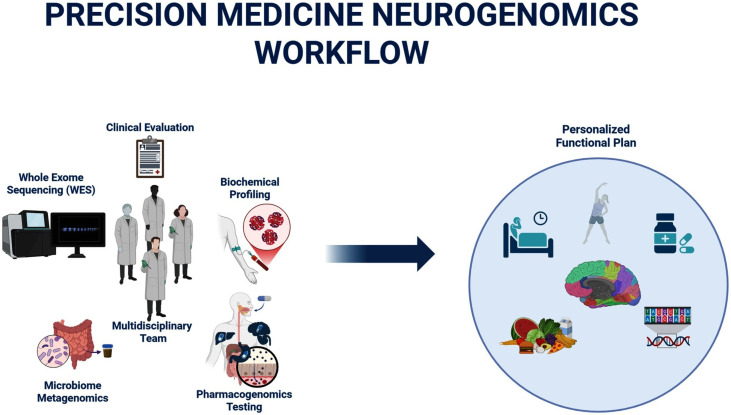
Integrative neurogenomics workflow. Schematic representation of the proposed neurogenomics workflow, illustrating how phenotype-based evaluation, genomic analysis, biochemical profiling, metagenomic assessment, and multidisciplinary interpretation converge to generate individualized mechanistic hypotheses. Created in https://BioRender.com

Combining genomic data, polygenic risk scores (PRS), and biochemical biomarkers constitutes the core of the integrative workflow (Figure 1). Ideally, this framework is further enriched with metagenomic analysis of the gut microbiome, which provides valuable insights for functional decision-making (Proctor et al., 2019), and with a pharmacogenomic profile, particularly in cases requiring pharmacotherapy or where patients are already on complex polypharmacy regimens. This is especially common in older adults, who often consult multiple organ-specific specialists—neurologists, gastroenterologists, endocrinologists—without cross-communication between them. As a result, patients may end up with 10–14 concurrent medications, prescribed without consideration of their metabolizer status. Gene–drug, drug–drug, and drug–nutrient interactions may involve mutual inhibition or induction of metabolic pathways, leading not only to potential adverse reactions, nullification or exacerbation of therapeutic effects, but in some cases to fatal outcomes, depending also on whether the compound is a prodrug or an active drug (Dorado et al., 2007; Roden et al., 2019; Bosó et al., 2013).

To illustrate the functional relevance of this workflow, we highlight one representative patient case. While the workflow has been systematically applied across multiple patients, this case exemplifies how exome sequencing, PRS calculation, and biochemical profiling can converge into a consolidated report and ultimately a targeted clinical plan.

Based on this report, the MDT committee integrated by the medical coordination team, the clinical geneticists, the bioinformaticians, the precision medicine and clinical genomics expert, the nutritionist trained in genetics, and the relevant phenotype-specific specialist, reviews the findings collaboratively. Each discipline contributes its perspective, ensuring that recommendations are not only evidence-based but also harmonized across specialties. This MDT model has already demonstrated efficacy in oncology, where molecular tumor boards are now integrating to standard practice (Ndoro, 2014; Taberna et al., 2020; Okasako and Bernstein, 2022). However, in neurodegeneration and systemic chronic disease, similar frameworks remain underutilized despite their potential for immediate clinical relevance.

Following consensus, the MDT designs a functional precision plan (Table 5), which provides specific recommendations aimed at restoring balance across key domains. These include tailored nutrition plans, personalized supplementation, exercise regimens, and—where necessary—pharmacological interventions adjusted for the patient’s genetic and metabolic background.

### 1.9 Clinical translation to treating specialist

Once consensus on diagnosis and treatment has been reached, a consultation is scheduled with the patient or their family (when applicable). During this meeting, the specialists ensure that the information is clearly understood and emphasize the need for strict adherence and discipline in following the recommendations—particularly those involving significant changes in diet and lifestyle—in order to achieve the best possible outcomes. A follow-up consultation and evolutionary analysis monitoring are then scheduled, typically at 3 months, focusing mainly on the parameters that were previously found to be altered.

In addition, our team conducts regular weekly telephone follow-ups, updating the patient’s clinical record with progress notes and remaining attentive to any changes, adjustments, or unexpected reactions in order to make timely modifications as needed.

The mechanistic rationale for integrating these layers is grounded in the multifactorial and polygenic biology of neurodegeneration. Whole-exome sequencing captures all coding regions, including both highly penetrant variants as well as incomplete penetrance variants that require to be classified following ACMG pathogenicity criteria; biologically-oriented common variant burden profiling reflects polygenic modulation of key pathways such as inflammation, lipid metabolism, mitochondrial function and synaptic integrity; biochemical markers provide a real-time phenotype of metabolic, infectious, hormonal, inflammatory and toxic states; and functional metagenomic data reveal microbial pathways known to influence immune, metabolism, neurotransmission, hormonal, amino acid synthesis, and resistome host status. When interpreted through a multidisciplinary clinical lens, these complementary layers converge to generate individualized, mechanistically informed hypotheses about etiological subtypes and potential therapeutic targets.

These pathways were selected because they correspond to the core biological foundations of neurodegeneration—proteinopathy, synaptic disfunction, neuroinflammation, mitochondrial impairment, vascular and metabolic dysregulation, among others—thereby anchoring the workflow in mechanisms that are central to dementias and making it applicable across AD, FTD, DLB, vascular and mixed dementias, as well as related neurodegenerative phenotypes that share these pathogenic mechanisms.

## 2 Highlights of illustrative case

This case is presented as an illustrative demonstration of how the multi-omic workflow operates in practice.

Male patient, +60 years old, who presents cognitive decline, episodic memory impairment, and executive dysfunction. Findings were initially compatible with dementia with Lewy bodies and frontotemporal dementia, diagnosed by positron emission tomography (PET). The neurodegenerative course evolved toward a presentation consistent with primary progressive aphasia.

### 2.1 Genomic findings

Genomic and bioinformatic analyses followed a standardized workflow including QiAamp DNA extraction, Illumina DNA Prep with Exome 2.5 Enrichment, and XLEAP-SBS sequencing at ~100 × coverage. Reads were processed using DRAGEN (<v4.0) with GRCh38, yielding 271,809 variants across 26,918 genes. Variants were annotated with VEP, dbNSFP, gnomAD, ClinVar, HGMD and related resources, classified using ACMG/AMP guidelines, and manually validated.

The polygenic component used a functional polygenic score (PRS-F), in which variants were weighted according to published effect sizes (OR/*β*) or validated functional evidence when quantitative estimates were unavailable. The weighted sum was normalized using a z-transformation to enable percentile interpretation. Full methodological details are provided in the Supplementary material.

Within this conceptual framework, common benign variants are considered as contributors to pathway-level burden when supported by functional evidence, and are incorporated into the PRS-F to capture cumulative pathway effects for mechanistic interpretation, without implying additive pathogenicity or diagnostic weight at the clinical level.

Relevant exome findings—including pathogenic, likely pathogenic, VUS, secondary and incidental variants—as well as the functional polygenic score (PRS-F) are summarized in Tables 2, 3, with the full expanded version provided in the Supplementary material.

### 2.2 Biochemical markers

The biochemical markers incorporated in this workflow are not intended as diagnostic biomarkers of dementia, but as mechanistic phenotypic layers that modulate neurodegenerative processes (e.g., mitochondrial stress, inflammation, lipid dysregulation, metal homeostasis, metabolic impairment) and interact with genomic and metagenomic findings to inform individualized therapeutic hypotheses. Whenever available, structural and functional neuroimaging (MRI, FDG–PET, amyloid or tau PET) is integrated into the multidisciplinary evaluation. The workflow, however, is designed to remain operational and mechanistically informative even when advanced neuroimaging is not accessible, reflecting real-world variability in diagnostic resources.

Key metabolic, inflammatory, hormonal, micronutrient, and toxicological biomarkers are presented in Table 4.

### 2.3 Functional precision plan

The interventions discussed in this case are not proposed as validated or disease-specific therapies, but as mechanistically informed hypotheses generated through integrative interpretation of genomic, biochemical, microbial and clinical findings. Only genomic and biochemical findings that mapped to well-characterized, mechanistically actionable pathways were considered in the formulation of the functional precision plan, with the aim of guiding interventions towards biologically coherent and clinically modifiable axes relevant to the patient’s phenotype. Their purpose is to illustrate how mechanistic interpretation of multi-omic findings can inform personalized therapeutic considerations.

The personalized intervention plan—including tailored nutrition, evidence-based supplementation, pharmacogenomic considerations (where applicable), and targeted lifestyle modifications—is detailed in Table 5. Follow-up of this case is currently ongoing. While long-term outcomes are not yet available, preliminary monitoring aims to assess early clinical responses and guide subsequent adjustments. These results will provide further evidence of the clinical utility of the integrative workflow.

## 3 Discussion

With the increasing prevalence of neurodegenerative diseases, their diagnostic and therapeutic complexity—driven by multifactorial and polygenic burden—and the limited success of traditional approaches, sustained skepticism remains regarding the prospect of a single “magic bullet” therapy. Conventional strategies for the management of neurodegenerative disorders have historically focused on symptomatic treatment. For decades, cholinesterase inhibitors in conjunction with NMDA receptor antagonists have remained the standard treatments for mild, moderate to severe stages of dementia (Wu and Fuh, 2025), more recently complemented by anti-amyloid immunotherapies. However, clinical improvements have remained modest, with little impact on disease trajectory. These approaches often treat isolated symptoms without addressing the molecular and metabolic dysregulation that underlies disease progression. Although lifestyle measures, vascular risk management, and cognitive therapies are acknowledged as essential, they are often applied in generic terms, without personalization or integration into a unified plan.

While no single pharmacological or integrative strategy can be universally effective, the aim is not to impose a standardized recipe, but to combine the most relevant interventions at the molecular, metabolic, and clinical levels for each patient.

In the illustrative case, we can highlight a significant metabolic component that was evident, with an elevated atherogenic index, increased homocysteine and insulin, markedly elevated B12 levels—due to exogenous supplementation as documented in the clinical history, elevated TSH, slightly elevated homocysteine, and moderately reduced vitamin D. Metabolic alterations may reflect not only lifestyle factors but also the possible influence of genetic variants such as *LRP8* (atherothrombotic processes, cardiovascular response), *CAPN10* (type 2 diabetes mellitus, atherosclerosis, insulin sensitivity and secretion), *FCN3* (innate immunity), *CHI3L1* (inflammation and neuroinflammatory pathways), *GABRA2* (anxiety and behavioral regulation), and *NPSR1* (anxiety, inflammatory responses). Additional findings included hormonal imbalances and elevated levels of heavy metals such as mercury and lead. Genomic analysis further suggested possible interaction with copper accumulation, indicating the need to monitor free copper and ceruloplasmin levels. Despite the proposed workflow including a metagenomic analysis of the intestinal microbiome as a very important part of diagnosing the potential influences or vulnerabilities that may impact the phenotype, it has not yet been possible to apply to this patient due to budgetary reasons. This incident is an example of the true barriers to implementing this model and underscores the need for scalable and budget-friendly solutions if frameworks like this one are to be implemented on a widespread basis.

It must be acknowledged that functional supplementation and lifestyle-based interventions appear most effective when introduced early, and that current evidence for efficacy in late-stage disease remains limited. Nevertheless, we argue that returning to the disrupted molecular and metabolic axes that initiate the pathology offers a more coherent strategy to achieve meaningful improvement and preserve quality of life. As illustrated in this case, targeted supplementation aligned with genomic risks and biochemical findings enables a personalized approach, selecting only the compounds required at the current stage and adjusting them dynamically as reference values evolve.

The proposed workflow contextualizes genomic data as a clinical tool rather than a test reserved for cases with strong family history or clear inheritance patterns. Recent evidence suggests that polygenic scores may even serve as diagnostic adjuncts in complex cases (McKeever et al., 2025; Zettergren et al., 2021). Integrating genomics, biochemical markers, and gut microbiome metagenomics into a functional workflow may represent a promising and potentially more comprehensive approach. The incorporation of a multidisciplinary team (MDT) allows for the integration of perspectives across specialties, reducing common sources of iatrogenesis, polypharmacy without inter-specialty communication, and professional discredit between disciplines—scenarios in which the patient is ultimately the one who suffers (Zaij et al., 2023; Sutanto, 2025).

Nevertheless, the proposed model requires validation in larger longitudinal cohorts and operates within the current limitations of the field, including the lack of standardization in variant filtering, classification, and polygenic risk score calculation.

As mentioned before, another major limitation is the current cost of incorporating multiple layers of analysis, which remains affordable to only a minority of patients in both the public and private sectors. Demonstrating positive outcomes in adequately powered longitudinal studies will be essential to support reimbursement by payers and insurers.

Another practical challenge in implementing this integrated neurogenomics workflow is the substantial human-resource coordination and technical integration required across sequencing, bioinformatics, laboratory, and clinical teams—factors that may limit scalability in many healthcare settings.

Another substantial barrier remains the incorporation of precision medicine and clinical genomics into medical school curricula. Until such integration occurs, continuing medical education—through courses, certificate programs, master’s degrees, and doctoral training—will be critical to upskill specialists and facilitate adoption in clinical practice, both for diagnosis and for treatment.

Finally, one of the greatest challenges the proposed model faces in achieving positive outcomes is ensuring strict adherence from patients and families to the multiple recommendations of the functional precision plan. This limitation is also shared by previous multidomain protocols, which have reported modest yet encouraging results in early cognitive impairment (Toups et al., 2022). In both cases, the main difficulty stems less from theoretical shortcomings than from the practical challenge of implementing coordinated interventions across numerous physiological axes. Building upon these earlier frameworks, the proposed model integrates additional biological layers—including advanced genomics, metagenomics, consolidated functional reporting, and the systematic involvement of a multidisciplinary team for diagnosis and treatment—which may enhance precision and clinical impact. This expanded approach—centered on the improvement of physiological markers through dietary and lifestyle modifications personalized to an individual’s genomic profile—has the potential to yield more substantial benefits in both the prevention and reversal of cognitive decline, though further validation in controlled studies is required.

The present framework should be understood as a conceptual, falsifiable model rather than a demonstration of clinical efficacy within a single case. As stated in the introduction, our central hypothesis is that an integrative neurogenomics workflow can generate individualized, testable diagnostic and therapeutic hypotheses by linking genomic findings, biochemical phenotypes, microbiome signatures and multidisciplinary clinical interpretation. This hypothesis can be evaluated prospectively against standard-of-care diagnostic pathways in future studies.

## 4 Future directions

### 4.1 Integration with AI and digital decision-support systems

In recent years, AI has been incorporated into medical decision-making workflows for both individual practitioners and for clinics, health centers, and platforms. This has led to reports of significantly higher precision and speed when it comes to suggesting diagnoses and treatments (Jeong et al., 2025; D’Adderio and Bates, 2025). While results will still need to be validated by specialists—who retain responsibility for decisions and recommendations—the paradigm of decision-making for both patients and clinicians is changing at an unprecedented pace. Similarly, the precision of in-silico analyses, calculations, and models is experiencing accelerating success at a rate that was previously unthinkable for any research laboratory (Dalbanjan et al., 2025; Raman et al., 2025; Kant et al., 2025).

### 4.2 Genotype-guided clinical trials and recruitment

Genotype-guided clinical trials and recruitment represent a critical future direction. A major limitation in many published studies at present, is the lack of systematic participant genotyping, which introduces a significant source of bias into results. Overcoming this gap as genomics become increasingly integrated into clinical research will be essential to improve trial design, interpretability, and therapeutic precision.

### 4.3 iPSC and organoid models for mechanistic validation

Although a great number of genetic variants have been identified in population studies (GWAS), these associations alone do not demonstrate the precise biological mechanism by which a genetic variant contributes to disease. Recently, induced pluripotent stem cells (iPSCs) and organoid models have demonstrated great value for the functional and mechanistic validation of genetic variants and their respective pathologies (Lancaster and Knoblich, 2014). iPSCs allow for the differentiation of neurons, astrocytes, and microglia from the same patient carrying the variant (Muffat et al., 2016). Furthermore, 3D models reproduce the interactions between different cell types, offering a physiological context rather than a simple isolated cell line (Quadrato et al., 2017). These models allow researchers to measure gene expression, proteomics, and metabolism, observe the accumulation of abnormal proteins (tau, prions, *α*-synuclein), and test drugs, among other things (Raja et al., 2016). The current limitations must be considered, primarily the challenge of translating a patient’s own epigenetic signature (Kim et al., 2010).

### 4.4 Cell and gene therapies

Cell and gene therapy is advancing at a rapid pace, offering great promise and results not only for neurodegenerative diseases but for all other medical specialties. Significant challenges remain concerning the type of therapy, the type of culture, the type of differentiation and route of administration—whether in-vivo or ex-vivo—required to achieve relevant and sustained changes in dysfunctional gene expression at either the genomic or transcriptomic level, e.g., *APOE2*-mimetic gene therapy (Jackson et al., 2024). This is without mentioning, of course, the major challenge of ethical considerations, the supervision and standardization of culture laboratories, and cell banks (in the case of allogeneic cell therapies), among many other aspects to consider.

### 4.5 Multicenter collaborations and data-sharing frameworks

The future of functional precision medicine in neurodegeneration will require multicenter collaborations that transcend individual institutions and national boundaries. Harmonized data-sharing frameworks are essential to enable the aggregation of genomic, biochemical, and clinical information at scale, while safeguarding privacy and ethical standards. Emerging federated bioinformatics platforms offer the possibility of real-time integration and analysis across sites, allowing specialists—whether in academic, public health, or private settings—to access standardized pipelines and decision-support tools (Alvarellos et al., 2023; Huremagic et al., 2025; National Human Genome Research Institute (NHGRI), 2021).

## 5 Conclusion

Neurodegenerative diseases continue to rise at an unprecedented rate, and current clinical models—largely reliant on pharmacological therapies—have so far failed to provide a definitive solution to the problem. Functional protocols that incorporate the assessment of biochemical markers and limited genetic testing have achieved modest success in preventing, and in some cases reversing, early cognitive impairment, by leveraging nutrition, supplementation, and lifestyle interventions to regulate multiple physiological axes, aiming to halt aggressors and restore homeostasis.

The proposed integrative neurogenomics workflow, despite all the limitations previously noted, seeks to enhance these functional protocols to the next level by incorporating advanced genomics, pharmacogenomics, and metagenomics as appropriate on a case-by-case basis, along with MDT support at every stage of the process—laying a possible foundation for a new personalized genomic medicine standard in neurodegeneration.

After all, the “one-size-fits-all” approach was never correct, for in the end, medicine was never meant to fit all, but to fit each one.

## Data Availability

The original contributions presented in the study are included in the article/supplementary material, further inquiries can be directed to the corresponding author.
