# Supplementary material for: An integrative neurogenomics workflow for precision medicine in neurodegenerative disorders
Source: Front Dement. 2026 Feb 4;5:1745504. doi: 10.3389/frdem.2026.1745504 (PMC12913181; doi:10.3389/frdem.2026.1745504)
Supplement: Supplementary file 1 [file Data_Sheet_1.pdf]

SUPPLEMENTARY METHODS

FUNCTIONAL POLYGENIC RISK SCORE (PRS-F)

Of all the variants reported, some have OR/β in formal published articles and others present functional but non-quantitative reproducible evidence, so a hybrid model was chosen where both elements were combined.

Weights were derived from a standard discretization scheme, typically employed when variant sets are limited in size or heterogeneous.

**Evidence**	**OR/β**	**PRS-F Weight**
Strong association	OR > 1.30 or β > 0.20 (replicated in ≥2 studies) and/or strong convergent functional evidence	3
Moderate association	OR 1.10–1.30 or β 0.10–0.20	2
Weak association	OR < 1.10 o β < 0.10	1
Protective association	OR < 1.0 or reverse effect	–2
Strong functional evidence without OR	Direct impact on protein expression or key pathways	2 or 3 depending on magnitude [1]
Weak functional evidence	Indirect effect, not replicated	1

[1] When quantitative effect sizes were unavailable, functional convergence was defined operationally as the presence of at least two independent lines of functional evidence pointing to the same biological mechanism. Variants meeting this criterion through replicated effects on gene or protein expression, or through well-characterized pathway-level involvement, were assigned a weight of 3. Variants supported by functional evidence that was mechanistically plausible but less directly replicated or less specifically mapped to a single pathway were assigned a weight of 2.

ANALYSIS BY VARIANTS

1. *GRN* rs5848 (TT): Weight = 3

Reported pooled odds ratios in the range of 1.23 to 1.24 (TT vs CC; recessive model) for neurodegenerative diseases, with higher effect sizes reported in FTLD-focused cohorts. This variant has been shown to reduce progranulin expression by approximately thirty to fifty percent, supporting a neuroinflammatory vulnerability pathway.

Category: Strong association plus direct functional evidence.

Assigned weight: 3

2. *CHI3L1* rs4950928 (CC): Weight = 3

This variant has been associated with Alzheimer's disease risk in a case–control cohort and shows concordant biomarker-level evidence, with the CC genotype linked to elevated plasma CHI3L1 (YKL-40) levels and greater disease severity. Given the established role of the CHI3L1/YKL-40 axis in neuroinflammatory processes, this variant was considered mechanistically relevant despite the absence of a single, universally reported effect size.

Category: Case–control association + biomarker-supported functional relevance.

Assigned weight: 3

3. *HFE* rs1799945 (p.His63Asp / H63D): Weight = 2

Large GWAS do not consistently link HFE-H63D directly to Alzheimer's disease; however, H63D carrier status has been discussed in relation to oxidative stress susceptibility and neurotoxic vulnerability. Clinical reports have described mild cognitive phenotypes in some carriers, although dementia-specific associations remain inconsistent across cohorts.

Assigned weight: 2

4. *ATP7B rs121907998* (p.Met645Arg): Weight = 2

Functional evidence indicates that heterozygous carriers may develop mild neurological manifestations in some contexts. Altered copper handling has been documented and is mechanistically linked to oxidative stress, which may contribute to neurodegenerative vulnerability.

OR/β: Not available from GWAS-based estimates.

In asymptomatic carriers, evidence of chronic neuronal injury or sustained oxidative stress may be indirect or limited.

Assigned weight: 2

5. *ABCA7* (rs3752246 / rs4147929 / rs3764650): Weight = 2

A meta-analysis (36 studies) reports OR ≈ 1.10–1.20 depending on population. Although these variants individually confer small-to-moderate effects, ABCA7 shows consistent locus-level association across multiple GWAS.

Assigned weight: 2

6. *LRP8* rs5174: Weight = 2

LRP8 encodes ApoER2, a core receptor in the Reelin signaling pathway, which plays a critical role in synaptic plasticity, learning, and memory. Functional studies have demonstrated that LRP8-regulated enhancer activity and ApoER2-mediated signaling contribute to synaptic homeostasis and neuronal resilience. Given the recognized contribution of vascular and synaptic dysfunction to vascular dementia and mixed Alzheimer's disease presentations, this locus was considered a plausible vascular–synaptic vulnerability modifier. Evidence remains primarily functional rather than disease-specific, and is therefore interpreted as vulnerability-modifying rather than a strong disease-defining association.

Assigned weight: 2

7. *APOE* rs440446 (promoter): Weight = 1

This is not an ε4 or ε2-defining variant. It has been reported to modulate APOE expression in specific haplotypic contexts, with small or non-significant effect sizes in most cohorts.

Assigned weight: 1

8. *NPSR1* rs324981: Weight = 1

This variant has been associated with psychiatric phenotypes and modulation of cognitive traits (including verbal memory) in some studies. Dementia-specific effect sizes are not robustly established.

Assigned weight: 1

9. *LRRK2* rs7133914 (p.Arg1398His): Weight = −2

Evidence from Parkinson's-related studies suggests a protective association (OR < 1.0), and functional data indicate reduced kinase activity. However, these findings are largely Parkinson's-focused rather than dementia- or Alzheimer's-specific, and extrapolation to broader neurocognitive risk is uncertain. Allele-frequency variability across populations may further influence the magnitude and generalizability of the reported effect, including in Hispanic/Latin American cohorts.

Assigned weight: −2

SUMMARY TABLE

Variant	OR/β usado	Type of evidence	Justification	Weight
GRN rs5848	OR 1.23–1.24 (TT vs CC; recessive model)	Meta-analysis + strong functional effect	Progranulin expression (reported reduction 30-50%)	3
CHI3L1 rs4950928	Reported association (effect size not explicitly quantified)	Case-control association + biomarker correlation	Case–control association with AD risk w/concordant biomarker evidence (CHI3L1/YKL-40 axis)[1]	3
HFE rs1799945	n/a	Functional	Increased oxidative stress susceptibility / redox vulnerability	2
ATP7B rs121907998	n/a	Functional	Altered copper homeostasis (dyshomeostasis)	2
ABCA7 rs3752246; rs4147929; rs3764650;	OR 1.10–1.20	GWAS + meta-analysis	Late-onset AD risk (locus level association)	2
LRP8 rs5174	Functional (no OR reported)	Synaptic/Reelin pathway; vascular synaptic modifier	Vascular–cognitive vulnerability	2
APOE rs440446	Small/inconsistent	Population genetics/ association	Mild expression modulator	1
NPSR1 rs324981	Not established	Psychiatry/Memory	Neuropsychiatric/verbal memory modulator	1
LRRK2 rs7133914	OR < 1.0	Case–control / consortium (PD)	Protective association (uncertain transferability to dementia)	–2

[1] A weight of 3 was assigned to variants with direct association estimates and strong pathway-level relevance to a core mechanistic domain (here, neuroinflammation), even when evidence is cohort-specific.

Variant weighting:

For each included genetic variant (i), an individual weight (wi) was assigned using a discretized weighting framework designed for functional polygenic models with heterogeneous evidence. Weight assignment was based on three criteria: (1) magnitude of reported association when available (e.g., odds ratios from GWAS or meta-analyses), (2) level of supporting evidence (e.g., GWAS, meta-analysis, case–control, or functional studies), and (3) functional relevance within the implicated biological pathway. This discretization strategy allows integration of variants for which quantitative effect sizes are not directly comparable and is commonly applied when the number of variants is limited or heterogeneous.

Score aggregation:

The functional polygenic risk score (PRS-F) was calculated as the sum of the individual variant weights across all included variants, expressed as:

PRS-F = Σ wi (for i = 1 to n)

In the present case, the following variants were included in the calculation:

GRN + CHI3L1 + ABCA7 + HFE + ATP7B + LRP8 + APOE + NPSR1 − LRRK2

Based on the assigned weights, the resulting raw PRS-F value for this individual was:

PRS-F = 14

Estimation of Reference Parameters

In the absence of a large-scale population reference cohort for this specific functional model, reference parameters were derived through a targeted estimation framework. This approach is tailored to the discrete nature of the scoring system, which utilizes a curated set of variants rather than genome-wide data.

The expected mean (μ) was established at 10. While the theoretical maximum for the current 9-variant set is 27, this baseline is derived from a probabilistic centering approach rather than a simple arithmetic midpoint. This calibration is justified by the following factors:

- Probabilistic Summation and Allelic Distribution: The mean represents the expected cumulative burden in a modeled population where risk and protective alleles are not uniformly distributed. By centering the distribution at 10, the model accounts for

the low statistical probability of an individual carrying the maximum weighted genotype across all heterogeneous loci.

- Targeted Functional Weighting: Unlike agnostic genome-wide models, this approach focuses on variants with established biological relevance. The mean of 10 serves as a "functional baseline," reflecting the cumulative impact observed in simulation-based approximations of individuals with average polygenic susceptibility.

- Model Scalability and Dynamic Centering: This methodology is designed to be adaptable. For larger datasets, the expected mean is operationally defined as a weighted central tendency. This value is subject to adjustment based on the specific prevalence of risk alleles in the target population and the distribution of assigned weights (wi), ensuring the Z-transformation remains calibrated to the architectural complexity of the gene set.

The standard deviation was established at $\sigma = 3$ for the current 9 variant iteration. To maintain the model's internal validity as more loci are integrated, this parameter is governed by the following principles:

- Evidence Heterogeneity: It captures the variance inherent in aggregating weights from disparate sources, such as GWAS-derived odds ratios and qualitative functional evidence.
- Proportional Dispersion: This setting accounts for the spread observed in discretized scores where the contribution of individual variants is weighted by biological relevance rather than a uniform distribution.
- Model Scaling (Variance Adjustment): In this framework, σ is calibrated to reflect approximately 25–30% of the mean's magnitude, a range consistent with discretized functional scoring systems. This ensures that the Z-transformation remains sensitive to the cumulative functional burden without being disproportionately influenced by the increased range of a larger gene set.

Normalization and Clinical Stratification

To contextualize the raw PRS-F within a comparative landscape, the score was standardized via z-transformation: $Z = (\text{PRS-F} - \mu) / \sigma$

Applying the patient's raw score of 14:

$Z = (14 - 10) / 3$

$Z = 4 / 3$

$Z \approx 1.33$

To bridge the gap between quantitative metrics and clinical utility, we utilized a classification scale based on the following burden tiers:

Burden Category	Z-score Range	Percentile Range
Low Burden	$Z < -0.84$	< 20th
Average Burden	$-0.84 \leq Z < 0.84$	20th – 79th
Moderate-to-High Burden	$0.84 \leq Z < 1.65$	80th – 94th
High / Critical Burden	$Z \geq 1.65$	≥ 95th

Interpretation

A Z-score of 1.33 indicates that the individual's functional polygenic burden lies 1.33 standard deviations above the estimated mean. This places the individual within the moderate-to-high burden category (approximately within the 80th–94th percentile range) according to the burden stratification framework, with interpretation centered on the lower portion of this range (around the 80th–85th percentile), reflecting the limited gene set analyzed.

This result is qualitatively interpreted as a moderate-to-high functional burden. While the score reflects a significant susceptibility— clearly exceeding the expected baseline functional load—it does not reach the extreme thresholds ($Z > 1.65$) associated with a critical genetic burden.

It is important to note that because this score is based on a limited set of variants (n=9) and discrete weighting (1, 2, 3), the underlying distribution is not perfectly continuous like a theoretical Gaussian curve. Therefore, the PRS-F serves as a mechanistic lens to contextualize genomic vulnerability at the pathway level. It functions as a clinical compass to navigate mechanistic vulnerability, rather than a deterministic diagnostic or a population-wide predictive risk score.

SUPPORTING BIBLIOGRAPHY

Chen, Y., Li, S., Su, L., Sheng, J., Lv, W., Chen, G., & Xu, Z. (2015). Association of progranulin polymorphism rs5848 with neurodegenerative diseases: a meta-analysis. Journal of neurology, 262(4), 814–822.

Fenoglio C, Galimberti D, Cortini F, Kauwe JS, Cruchaga C, Venturelli E, Villa C, Serpente M, Scalabrini D, Mayo K, Piccio LM, Clerici F, Albani D, Mariani C, Forloni G, Bresolin N, Goate AM, Scarpini E. Rs5848 variant influences GRN mRNA levels in brain and peripheral mononuclear cells in patients with Alzheimer's disease. J Alzheimers Dis. 2009;18(3):603-12. doi: 10.3233/JAD-2009-1170.

Bagyinszky, E., & An, S. S. A. (2025). Targeting Granulin Haploinsufficiency in Frontotemporal Dementia: From Genetic Mechanisms to Therapeutics. International Journal of Molecular Sciences, 26(20), 9960. https://doi.org/10.3390/ijms26209960

Dai, Qi-He, and Dao-Kai Gong. "Association of the Polymorphisms and Plasma Level of CHI3L1 with Alzheimer's Disease in the Chinese Han Population: A Case-Control Study." Neuropsychobiology, vol. 77, no. 1, Nov. 2018, pp. 29–37, doi:10.1159/000492536.

Liu, Yiting et al. "Mutant HFE H63D protein is associated with prolonged endoplasmic reticulum stress and increased neuronal vulnerability." *The Journal of biological chemistry* vol. 286,15 (2011): 13161-70. doi:10.1074/jbc.M110.170944

Wint Nandar, Elizabeth B. Neely, Erica Unger, James R. Connor, A mutation in the HFE gene is associated with altered brain iron profiles and increased oxidative stress in mice, Biochimica et Biophysica Acta (BBA) - Molecular Basis of Disease, Volume 1832, Issue 6, 2013, Pages 729-741, ISSN 0925-4439, https://doi.org/10.1016/j.bbadis.2013.02.009

Foruny Olcina, J. R., & Boixeda de Miquel, D.. (2010). Enfermedad de Wilson. Revista Española de Enfermedades Digestivas, 102(1), 53-54. Recuperado en 09 de diciembre de 2025, de http://scielo.isciii.es/scielo.php?script=sci_arttext&pid=S1130-01082010000100009&lng=es&tlng=es

Liu, D., Zhang, H., Liu, C., Liu, J., Liu, Y., Bai, N., Zhou, Q., Xu, Z., Li, L., & Liu, H. (2024). Systematic review and meta-analysis of the association between ABCA7 common variants and Alzheimer's disease in non-Hispanic White and Asian cohorts. Frontiers in aging neuroscience, 16, 1406573. https://doi.org/10.3389/fnagi.2024.1406573

Hollingworth, P., Harold, D., Sims, R., Gerrish, A., Lambert, J. C., Carrasquillo, M. M., Abraham, R., Hamshere, M. L., Pahwa, J. S., Moskvina, V., Dowzell, K., Jones, N., Stretton, A., Thomas, C., Richards, A., Ivanov, D., Widdowson, C., Chapman, J., Lovestone, S., Powell, J., … Williams, J. (2011). Common variants at ABCA7,

MS4A6A/MS4A4E, EPHA1, CD33 and CD2AP are associated with Alzheimer's disease. Nature genetics, 43(5), 429–435. https://doi.org/10.1038/ng.803

Lambert, J. C., Ibrahim-Verbaas, C. A., Harold, D., Naj, A. C., Sims, R., Bellenguez, C., DeStafano, A. L., Bis, J. C., Beecham, G. W., Grenier-Boley, B., Russo, G., Thorton-Wells, T. A., Jones, N., Smith, A. V., Chouraki, V., Thomas, C., Ikram, M. A., Zelenika, D., Vardarajan, B. N., Kamatani, Y., … Amouyel, P. (2013). Meta-analysis of 74,046 individuals identifies 11 new susceptibility loci for Alzheimer's disease. Nature genetics, 45(12), 1452–1458. https://doi.org/10.1038/ng.2802

Reitz, C., Jun, G., Naj, A., Rajbhandary, R., Vardarajan, B. N., Wang, L. S., Valladares, O., Lin, C. F., Larson, E. B., Graff-Radford, N. R., Evans, D., De Jager, P. L., Crane, P. K., Buxbaum, J. D., Murrell, J. R., Raj, T., Ertekin-Taner, N., Logue, M., Baldwin, C. T., Green, R. C., … Alzheimer Disease Genetics Consortium (2013). Variants in the ATP-binding cassette transporter (ABCA7), apolipoprotein E ϵ4,and the risk of late-onset Alzheimer disease in African Americans. JAMA, 309(14), 1483–1492. https://doi.org/10.1001/jama.2013.2973

Telese, Francesca et al. "LRP8-Reelin-Regulated Neuronal Enhancer Signature Underlying Learning and Memory Formation." Neuron vol. 86,3 (2015): 696-710. doi:10.1016/j.neuron.2015.03.033

Werthmann, Gordon C.*; Herz, Joachim*. Apoer2/Lrp8: the undercover cop of synaptic homeostasis. Neural Regeneration Research 19(12):p 2563-2564, December 2024. | doi: 10.4103/NRR.NRR-D-23-02002

Limon-Sztencel, A., Lipska-Ziętkiewicz, B. S., Chmara, M., Wasag, B., Bidzan, L., Godlewska, B. R., & Limon, J. (2016). The algorithm for Alzheimer risk assessment based on APOE promoter polymorphisms. Alzheimer's research & therapy, 8(1), 19. https://doi.org/10.1186/s13195-016-0187-9

Lennertz, L., Quednow, B. B., Schuhmacher, A., Petrovsky, N., Frommann, I., Schulze-Rauschenbach, S., Landsberg, M. W., Steinbrecher, A., Höfels, S., Pukrop, R., Klosterkötter, J., Franke, P. E., Wölwer, W., Gaebel, W., Häfner, H., Maier, W., Wagner, M., & Mössner, R. (2012). The functional coding variant Asn107Ile of the neuropeptide S receptor gene (NPSR1) is associated with schizophrenia and modulates verbal memory and the acoustic startle response. The international journal of neuropsychopharmacology, 15(9), 1205–1215. https://doi.org/10.1017/S1461145711001623

Heckman, M. G., Elbaz, A., Soto-Ortolaza, A. I., Serie, D. J., Aasly, J. O., Annesi, G., Auburger, G., Bacon, J. A., Boczarska-Jedynak, M., Bozi, M., Brighina, L., Chartier-Harlin, M. C., Dardiotis, E., Destée, A., Ferrarese, C., Ferraris, A., Fiske, B., Gispert, S., Hadjigeorgiou, G. M., Hattori, N., … Genetic Epidemiology Of Parkinson's Disease (GEO-PD) Consortium (2014). Protective effect of LRRK2 p.R1398H on risk of Parkinson's

disease is independent of MAPT and SNCA variants. Neurobiology of aging, 35(1), 10.1016/j.neurobiolaging.2013.07.013.

Gopalai, A. A., Lim, J. L., Li, H. H., Zhao, Y., Lim, T. T., Eow, G. B., Puvanarajah, S., Viswanathan, S., Norlinah, M. I., Abdul Aziz, Z., Lim, S. K., Tan, C. T., Tan, A. H., Lim, S. Y., Tan, E. K., & Ahmad Annuar, A. (2019). LRRK2 N551K and R1398H variants are protective in Malays and Chinese in Malaysia: A case-control association study for Parkinson's disease. Molecular genetics & genomic medicine, 7(11), e604. https://doi.org/10.1002/mgg3.604
